# Avian malaria in a feral-pet pigeon: a case report

**DOI:** 10.1186/s12936-024-05116-5

**Published:** 2024-10-02

**Authors:** Gillian Muchaamba, Kannan Venugopal, Bettina Gächter, Barbara Vogler, Udo Hetzel, Sarah Albini, Matthias Marti

**Affiliations:** 1 Institute of Parasitology, Vetsuisse and Medical Faculty, University of Zurich, Zurich, Switzerland; 2 Wellcome Centre for Integrative Parasitology, University of Glasgow, Glasgow, UK; 3 Graduate School of Cellular and Biomedical Sciences, University of Bern, Bern, Switzerland; 4 Section for Poultry and Rabbit Diseases (NRGK), Institute for Food Safety and Hygiene, Vetsuisse Faculty, University of Zurich, Zurich, Switzerland; 5 Institute of Veterinary Pathology, Vetsuisse Faculty, University of Zurich, Zurich, Switzerland

**Keywords:** *Columba livia forma domestica*, Avian malaria, *Plasmodium relictum*, Histopathology, Heat shock protein 70

## Abstract

**Background:**

Avian malaria is caused by diverse parasite species of the genus *Plasmodium,* and it affects various bird species. The occurrence of this disease in some wild bird species is sparsely documented due to the scarce availability of samples. Hence the pathogenicity in some hosts is not completely known. In addition, feral birds may act as reservoirs bridging the transmission cycle from wild migratory birds to domestic and zoo-kept bird species.

**Case presentation:**

An owner of pigeons adopted a feral pigeon (*Columba livia forma domestica*) and housed it together with his other pet-pigeons. The bird died unexpectedly a few weeks after a surgical procedure and necropsy revealed a severely anaemic carcass, with pale organs and hydropericardium. Histopathologic analysis revealed inflammatory infiltrates in the lung and liver, and monocytes and Kupffer cells contained haemozoin pigment indicative of phagocytosis of *Plasmodium-*infected erythrocytes. A high erythrocytic infection rate of 18% was evident in tissues and blood vessels in various organs. Furthermore, the thyroid had masses classified as thyroid carcinomas. Immunohistochemistry with anti- *Plasmodium falciparum* HSP70 antibody revealed positive signals in erythrocytes and intravascular leucocytes. Further microscopy analysis using a Hemacolor-stained impression smear revealed a high parasitaemia with an asynchronous infection showing all erythrocytic stages. Molecular diagnosis by PCR identified *Plasmodium relictum*, lineage GRW11 as the aetiological agent. The bird presented died most likely due to an acute infection as evidenced by the high blood parasitaemia, leading to major erythrocyte destruction. Further analyses of feral pigeons (n = 22) did not reveal any additional cases of *Plasmodium* infections.

**Conclusion:**

This study reports the first mortality associated with *P. relictum* lineage GRW11. The study supports previous studies, suggesting that *Plasmodium* infections are not frequent in pigeons. Host conditions like immunosuppression due to the tumour may have influenced the infection outcome in this fatal case. Use of anti-*P. falciparum* HSP70 antibody for detection of *P. relictum* antigens for immune assays in blood and tissue samples will be a useful tool for future studies.

**Supplementary Information:**

The online version contains supplementary material available at 10.1186/s12936-024-05116-5.

## Background

Across Europe, there is an alarming reduction in bird populations due to habitat changes and emerging diseases [1]. However, some bird species have adapted to live in close proximity to humans in urban and peri-urban areas. For example, feral pigeons (*Columba livia forma domestica*) are thriving with an estimated population of 17–28 million pairs in Europe and 20,000 to 25,000 breeding pairs in Switzerland [2, 3]. These birds have become a major nuisance, especially in cities where no control system is in place and overfeeding by well-meaning people leads to a population increase. Problems include noise pollution, deterioration of architectonic and urban heritage through soiling with their droppings and disease transmission [4]. Therefore, various countries manage the feral pigeon population in cities by banning the public from feeding the birds, controlled breeding by egg replacement or male sterilization and use of mechanical repellents.

Feral pigeons are known for carrying various pathogens, some of which are zoonotic such as *Chlamydia psittaci, Salmonella* spp*., Cryptococcus neoformans* and the parasite *Toxoplasma gondii* [5–7]. Haemosporidia are also common parasites infecting pigeons, namely protozoans in the genera *Haemoproteus, Leucocytozoon* and *Plasmodium*. *Haemoproteus columbae,* transmitted by hippoboscid ‘louse’ flies, causing haemoproteosis is the most common haemoparasite isolated from pigeons worldwide [8, 9]. Prevalence of haemoproteosis in pigeon populations is variable from 27.6% to 100% and is reportedly lethal to young pigeons [10–14]. Leucocytozoonosis caused by parasites of the genus *Leucocytozoon* has a prevalence of 15.7% in pigeons, according to a study from Italy [10]. Less frequently, previous publications have reported *Plasmodium relictum* infections in pigeons, with one study confirming occurrence of lineage SGS1 in one out of 15 haemosporidian positive pigeons [10, 15–17]. However, these few epidemiological studies did not focus on the pathological features caused by this *P. relictum* lineage. Despite the low detection rate of *P. relictum* infections in pigeons, the high feral pigeon population may pose a significant problem, as these birds may act as reservoir hosts for other more vulnerable bird species.

*Plasmodium relictum* is a globally distributed invasive parasite, infecting a vast range of bird species [18]. Effects of this parasite can vary from low transient parasitaemia to acute infection with high parasitaemia, subsequent anaemia and sometimes fatal outcomes. Outcomes depend on the host species and its adaptability to parasite infection [19, 20]. Based on the partial mitochondrial DNA sequence of the *cytochrome b* gene, five haplotypes/lineages have been described in *P. relictum*; namely pSGS1, pGRW11, pGRW4, pPHCOLO01, and pLZFUS01 [20]. Of these lineages, SGS1 and GRW11 are frequently reported in various bird species and vectors in Europe [18, 21–23]. In Switzerland, cases of *P. relictum* SGS1 infections have been reported in Great tits (*Parus major*), and in various zoo kept bird species including Superb starlings (*Lamprotornis superbus)* [24, 25] and fatal cases in African penguins (*Spheniscus demersus*) and Atlantic puffins (*Fratercula arctica)* [26]. *Culex pipiens* is a natural vector for *P. relictum,* as initially demonstrated in experimentally infected mosquitos in 1898 by Ronald Ross and other authors [27, 28]. In south-western Switzerland, identification of GRW11-positive mosquitoes was reported [25].

This report describes the occurrence of avian malaria caused by *P. relictum* GRW11 in a feral-pet pigeon and the first use of anti- *Plasmodium falciparum* HSP70 antibody to detect *P. relictum* antigens in infected birds.

### Case

#### History

An owner of pet pigeons in Sallanches, France, close to the border to Italy and Switzerland, had a wild pigeon (*Columba livia forma domestica*) repeatedly loitering on his windows. Eventually, he adopted the bird, named it “Puffo” and housed it with his pet pigeons. The bird was not presented to a veterinarian for a health check. Further, he brought Puffo to his second home in the canton of Valais, Switzerland. Because of swelling around one eye, it was admitted to a private veterinary practice in the canton of Geneva, where it underwent surgery to resect a peribulbar tumour. After surgery, the bird received daily treatments with eye-drops for two to three weeks until it died unexpectedly a few days later.

### Methods

#### Necropsy and histopathology, immunohistochemistry

The pigeon was submitted for necropsy to the Section for Poultry and Rabbit Diseases (NRGK), Vetsuisse Faculty, University of Zurich, to determine the cause of death. Sections of liver, lung, adrenal gland, trachea, brain, muscle, kidney, gizzard, and pancreas were fixed in buffered formalin for 24 h before being processed for routine histopathological analysis.

For avian malaria immunohistochemistry the protocol was adapted from previous studies in human malaria [29]. Formalin fixed, paraffin embedded sections (2 µm) were mounted on positively charged slides, dried overnight at 37 °C, deparaffinized in four xylele baths for five minutes and rehydrated in degressive ethanol series (100%, 90%, 70%) using the Prisma machine (DAKO Omnis, Dako Colorado, Inc., USA). Thereafter, the sections were transferred to EDTA-buffer, kept in a pressure cooker for 20 min at 98 °C, rinsed with distilled water and left in TBS-Tween buffer. Sections were then incubated with anti-*P. falciparum* HSP70 (Antibodies-online GmbH, Germany) at a dilution of 1: 300 for 60 min at room temperature, blocked with peroxidase blocking solution (Agilent Dako, USA) for 10 min at room temperature, rinsed, incubated with Envision + System HRP-conjugated secondary anti-rabbit antisera (Agilent Dako, USA) for 30 min at room temperature, and stained with DAB (3,3′-Diaminobenzidine) chromogen. Haematoxylin was used as a counterstain and consecutively, the sections were dehydrated in the Prisma machine with differing xylele concentrations (70%, 95%, 100%) and then covered with Tissue-Tek-Film (Sakura Finetek, USA). Sections of in-vitro derived *P. falciparum* blood clots served as a positive control, while tissue sections of PCR negative birds of various species served as negative controls to rule out false-positive reactions within different organs.

### Parasitology tests

Since the bird was severely anaemic, it was not possible to aspirate blood from the heart. Therefore, a blood clot was collected, and impression smears were made. One smear was fixed in methanol and stained with Hemacolor Rapid (Sigma-Aldrich, Merck, Schaffhausen Switzerland) for microscopic examination of parasites. The parasitaemia was determined by counting the number of infected red blood cells as a percentage of a total of 1000 red blood cells. Another smear was fixed in 4% paraformaldehyde and 0.0075% Glutaraldehyde, permeabilized in 0.1%TritonX-100 and then blocked in 3% Bovine Serum Albumin to be used for antigen detection in an immunofluorescence assay as previously described [30]. Rabbit anti-*P. falciparum* HSP70 antibody was used as a primary antibody at a dilution of 1: 500. After an incubation and wash step, a conjugated secondary antibody, Alexa Fluor594 anti-rabbit (1:1000 dilution) was added. Following incubation of the conjugated antibody, and washing, a cover slip was mounted with Vectashield anti-fade mounting medium containing DAPI (H1200-10- Vector Laboratories, Newark- CA, USA).

DNA was isolated from initially frozen samples of liver, lung, spleen, heart, adrenal gland, brain, and pectoral muscle using a DNeasy Blood & Tissue Kit (Qiagen, Hilden-Germany). A genus-specific multiplex PCR for avian haemosporidian parasites was used to screen the organs as described [31]. Thereafter, a nested PCR targeting the partial *cytochrome b* gene of *Plasmodium*, *Haemoproteus* and *Leucocytozoon* as previously described [32] was used on positive samples to allow sequencing for species and lineage determination using 80 ng of genomic DNA. The tissue PCR was run with each organ being analysed separately. Positive controls included genomic DNA of *Plasmodium matutinum* isolated from a Humboldt penguin, and genomic DNA from a coinfection of *Haemoproteus syrnii* and *Leucocytozoon* sp. in a tawny owl, while blood from an uninfected bird was used as a negative control. The success of the PCR was determined by the amplification of PCR amplicons with the positive controls and absence of false positives in the negative birds on a 2% agarose gel using electrophoresis. Positive PCR amplicons were purified using a Qiagen PCR Purification kit (Qiagen, Hilden-Germany) and sent for sequencing at Microsynth AG. The sequences were aligned, trimmed, and compared against reference sequences of avian *Plasmodium* and *Leucocytozoon* species obtained from the MalAvi database (http://130.235.244.92/Malavi/) accessed on 05.01.24, and from the NCBI database using Geneious Prime software.

### Bacteriology and PCR tests

To rule out *Chlamydia psittaci* and Pigeon *Paramyxovirus* Type 1 (PPMV-1), PCR was performed from a liver/spleen pool, kidney, and brain [33, 34]. A bacteriological examination from the liver and intestines was performed according to EN ISO 6579–1/A1:2020 to rule out *Salmonella* sp. [35]. Liver and lung were cultured on Columbia agar with 7% sheep blood and bromothymol blue lactose agar (Thermo Fisher Scientific, Waltham, USA) at 37 °C for 48 h. Mycobacteriosis was ruled out by microscopic evaluation of Ziehl–Neelsen staining of smears from the liver, adrenal gland, thyroid gland, and a cranial mass on the trachea.

### Further screening of feral pigeons in Zürich, Switzerland

To further investigate the presence of avian *Plasmodium* parasites in pigeons, city pigeons caught by the game warden as part of a population control programme by the City of Zurich were screened for parasites in a small trial between June and September 2023. These included two juvenile and twenty adult birds. The birds were dissected within 6 h of death and fresh organs (spleen, liver, heart, and lung) were harvested, frozen and later screened for haemoparasites using a nested PCR [32].

## Results

### Gross pathology

**Fig. 1:**
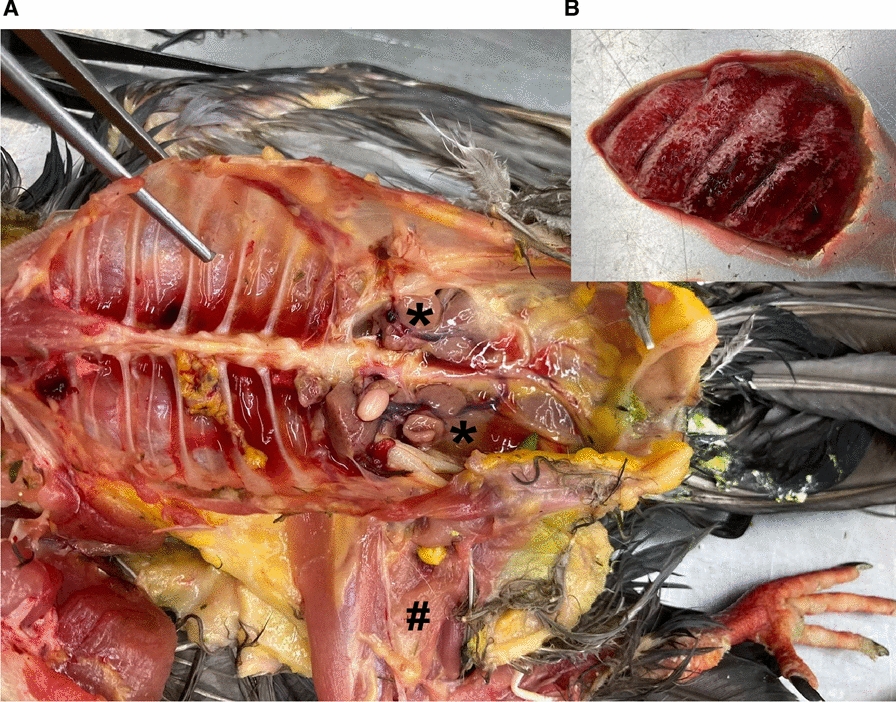
Gross pathology examination of a feral pigeon. **A**. A severely anaemic carcass with pale kidneys (*) and pale musculature (#). View of the coelomic cavity from cranial left to caudal right. **B**. Highly congested oedematous lung

Upon necropsy, the feral-pet pigeon was found to be severely anaemic, with muscle tissue, liver, kidneys, testicles, lungs, and other organs appearing pale in colour (Fig. 1A, B). The lungs were mildly congested, and the pericardium was filled with yellow serous fluid. Several healed ulcerations in the gizzard were observed. Both thyroid glands were severely enlarged (approx. 3 × 1 × 0.5 cm), each of them with 2–5 yellow foci (approx. 0.1 × 0.1 × 0.1 cm) inside. Furthermore, two masses were found attached to the outside of the cranial third of the trachea, without connection to the tracheal lumen. They were located 0.5–1 cm from each other. The cranial mass (approx. 1.2 × 1 × 0.5 cm) was of heterogenic consistency with multiple yellow foci, whereof some had calcified centres, and at least one cystic cavern containing yellow serous fluid. The distal mass (approx. 0.7 × 0.5 × 0.5 cm) neither showed caverns nor calcification, but also multiple yellow foci. No intestinal parasites were found.

### Parasitology tests

**Fig. 2:**
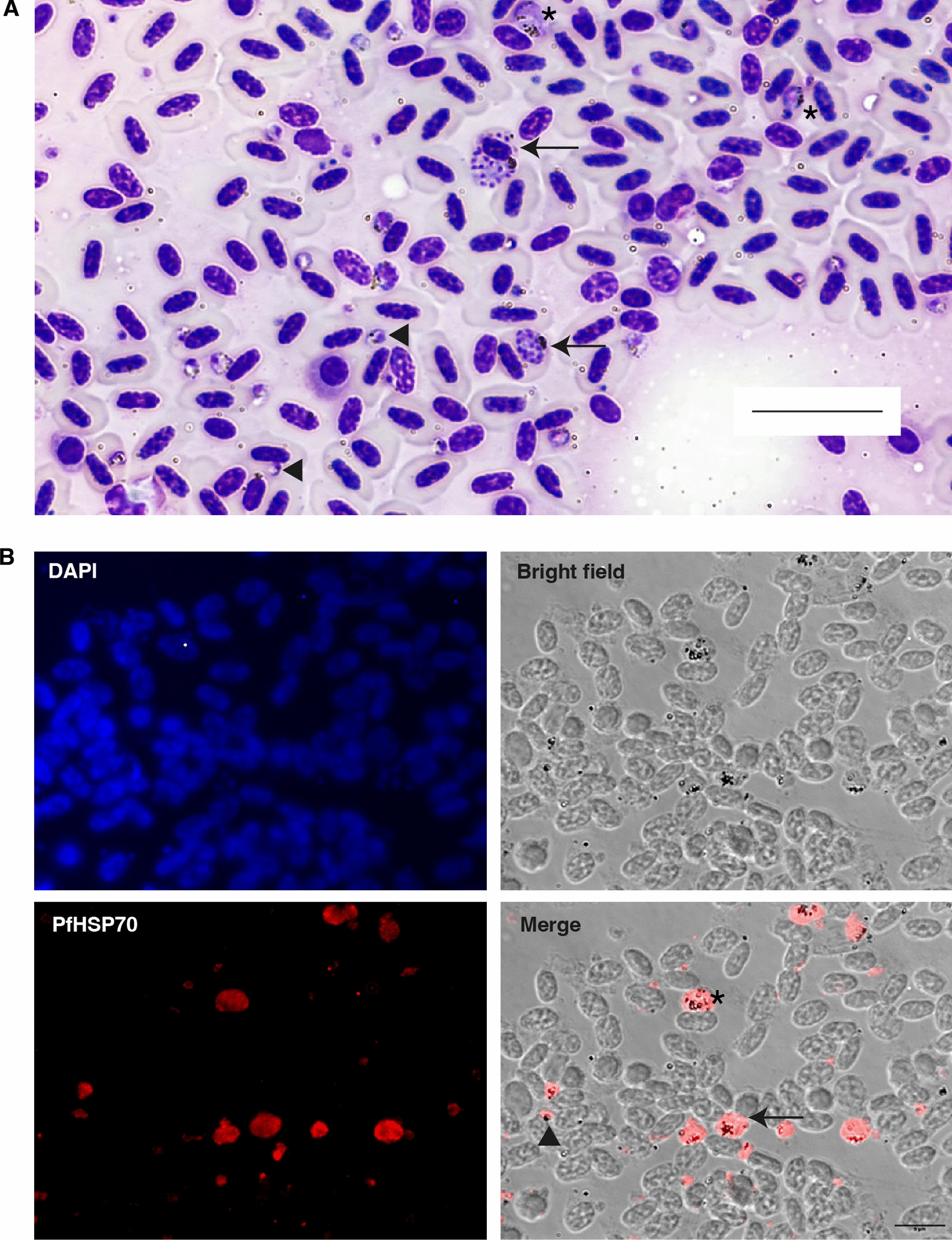
Analysis of impression smears. **A**. Hemacolor rapid staining of an impression smear from a blood clot. Shown is a representative field of view with infected red blood cells containing trophozoites (arrowheads), erythrocytic meronts (arrow) and gametocytes (asterisk). Haemozoin pigment is evident as a golden-brown structure in mature trophozoites, erythrocytic meronts and gametocytes. Scale bar: 20 μm. **B**. Immunofluorescence assay of an impression smear from a blood smear. DAPI panel shows stained red blood cell and parasite nuclei. The bright field panel shows the same representative view of red blood cells. Some infected red blood cells are evident with haemozoin pigments as black dots. *Pf*HSP70 and merge panel shows the positive reactions in infected red blood cells evident with red staining. Trophozoites (arrowhead) and erythrocytic meronts (arrow) have a center of accumulation of the haemozoin pigment, while in gametocytes (asterisk) the haemozoin pigment is scattered. Staining was done with anti-*P. falciparum* HSP70 antibody (1:500), secondary antibody anti-rabbit Alexa-Fluor 594 (1:1000) and DAPI. Scale bar: 9 μm

Microscopic examination of Hemacolor rapid-stained impression smears revealed an asynchronous infection of haemoparasites (Fig. 2A). Round and irregularly shaped trophozoites were seen and mature trophozoites displaced the host erythrocyte nucleus. Erythrocytic meronts containing up to 18 merozoites, with characteristic clumped-up haemozoin pigment were seen. Round gametocytes with scattered haemozoin pigment were present in the blood film and most displaced the host erythrocyte nucleus (Fig. 2A). The parasitaemia was determined to be approximately 18%. Immunofluorescence assay using anti-*P. falciparum* HSP70 antibody revealed red immunopositive reactions corresponding to different parasite stages (Fig. 2B). The antibody staining was diffusely distributed in the parasite cytoplasm. All organs screened with the haemosporidian multiplex and nested PCR were positive for *Plasmodium* species and the sequencing revealed *Plasmodium relictum* GRW11 infection, accession number PQ197205. The adrenal gland had a coinfection with *Leucocytozoon* spp. AEMO02, accession number PQ197206.

### Histopathologic analysis

**Fig. 3:**
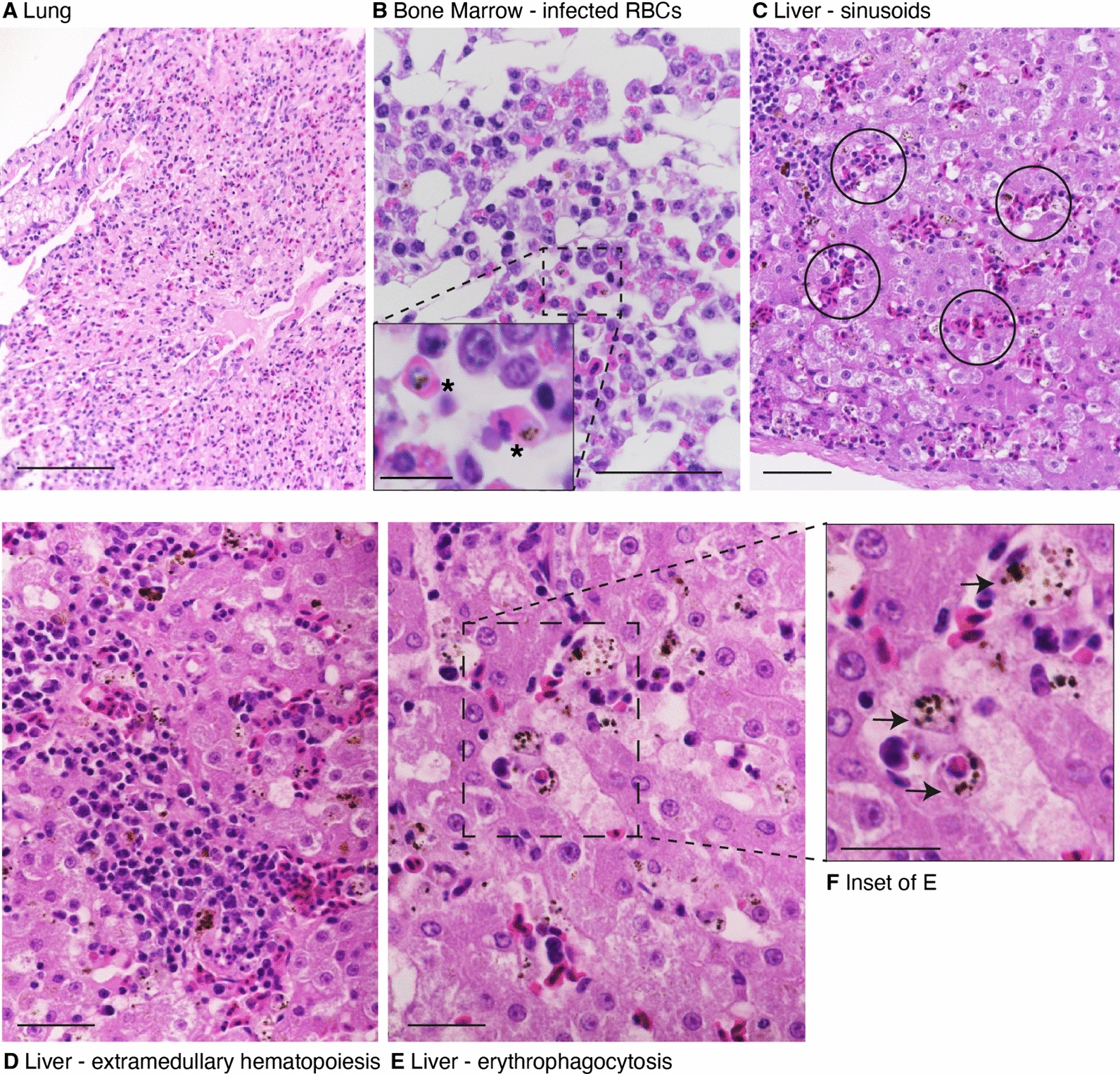
Histopathology (hematoxylin and eosin staining, H&E) of organs. **A**. Lung panel shows atelectasis with collapsed terminal respiratory units. Scale bar: 100 μm. **B**. Bone marrow shows evidence of infected red blood cells (RBC). Scale bar: 50 μm. Inset: Zoom in view of B showing two infected RBCs (asterisk). The left erythrocyte contains an intracellular gametocyte that has enucleated the host nucleus, while the right bottom RBC contains an erythrocytic meront that has laterally displaced the RBC nucleus. Scale bar 10 μm. **C**. Liver panel showing sinusoidal congestion (circles). Scale bar: 50 μm. **D**. Evidence of macrophages (Kupffer cells) with brown pigment, indicative of engulfed erythrocytes with haemozoin plus evidence of extramedullary haematopoiesis in the liver. **E**. Kupffer cells in the liver with haemozoin indicative of engulfed infected erythrocytes. Inset: Detail of E depicting macrophages with engulfed infected erythrocytes (arrows). Scale bar in D-F: 20 μm

Microscopic analysis revealed in various organs including the liver, lung, bone marrow, brain, pancreas, kidney, and thyroid, evidence of *Plasmodium* infection in erythrocytes with apparent haemozoin pigments and the parasite pushing the nucleus laterally (Fig. 3B). Furthermore, the lung revealed moderate acute parenchymal hyperaemia with focally atelectatic areas (Fig. 3A). There was moderate infiltration by heterophilic granulocytes and focally mild interstitial fibrosis. The liver showed moderate sinusoidal hyperaemia, moderate extramedullary haematopoiesis, mild hepatocellular lipidosis, and focally hepatocellular single-cell necrosis (Fig. 3C, D). Some Kupffer cells and monocytes contained round basophilic staining structures with nuclei indicative of phagocytosed infected erythrocytes containing merozoites. Pigment-laden erythrocytes, monocytes and macrophages were seen in various organs (Fig. S1 A, B, C and F) but were severe in the liver and lung and seen in almost every field of view (Fig. 3E and inset). The thyroid showed lymphoplasmacytic inflammatory infiltrates and masses classified as thyroid carcinomas which were not further characterized histologically (Figs. S1D and S1E). In the adrenal gland, the medulla consists of nodules with largely regular adrenal medullary cells (nodular hyperplasia) also with minimal interstitial lymphoplasmacytic as well as heterophilic infiltrates. The gizzard showed focal ulceration with adjacent low- to moderate-grade infiltration by lymphocytes, plasma cells, heterophilic granulocytes, and moderate fibrosis. Sections of the pancreas revealed low-grade interstitial lymphoplasmacytic infiltrates. The remaining organs were histologically unremarkable.

**Fig. 4:**
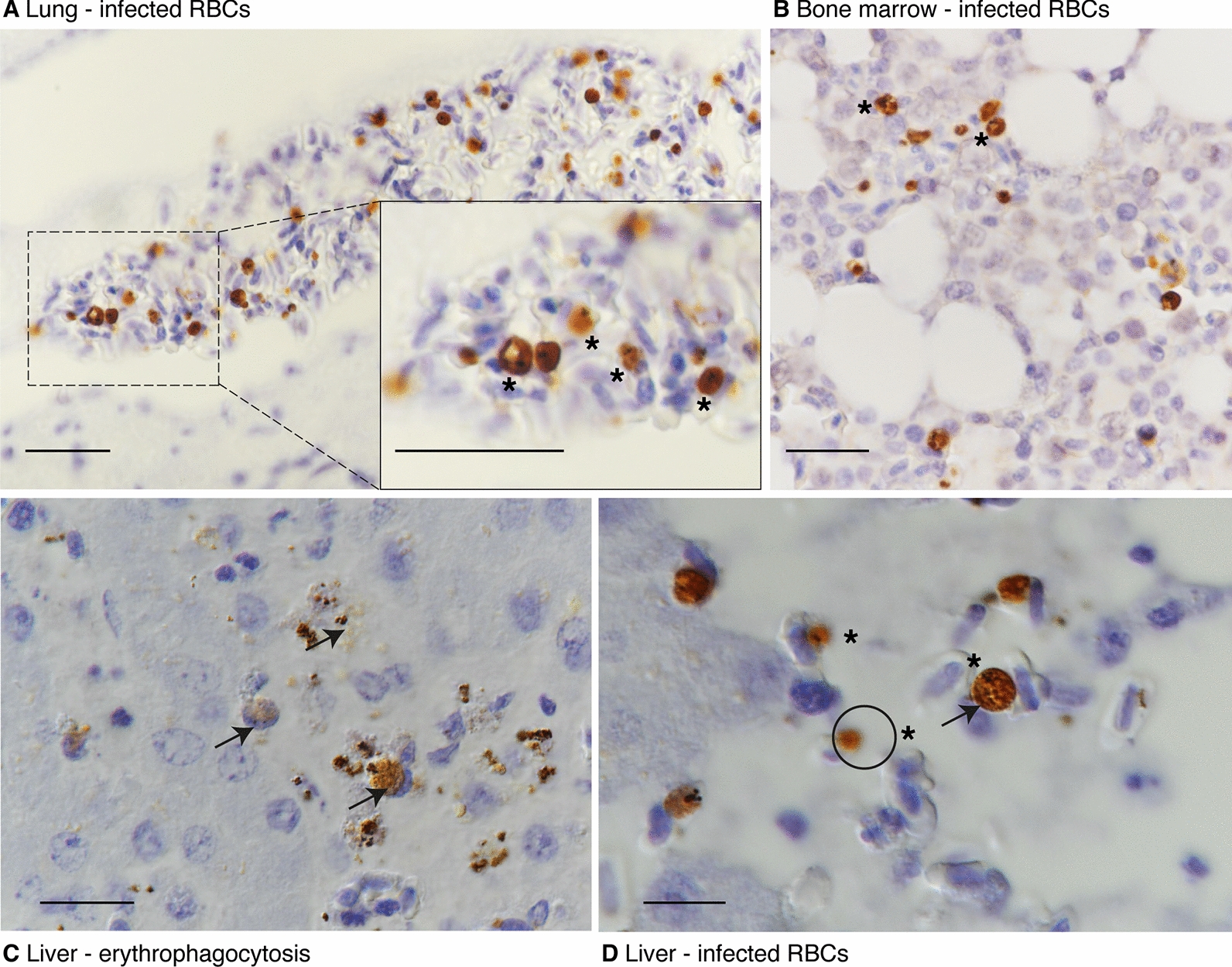
Immunohistochemistry. **A**. Lung blood vessel showing infected red blood cells (asterisk) diffusely stained brown with black haemozoin pigment. **B**. Bone marrow showing infected red blood cells. Scale A and B 30 μm. **C.** Liver section with positive monocyte/ Kupffer cell (arrows) and evidence of erythrophagocytosis in other monocytes evident as the remaining haemozoin pigments. **D**. Liver section depicting infected red blood cells at different stages of maturation. Shown is an immature trophozoite (circle) which is smaller and lacks haemozoin pigment (black spots), while other parasites stain dark brown and have haemozoin pigment (schizonts, arrow). **C** and **D** Scale bar 10 μm. Immunohistochemistry staining using Rabbit anti-*P. falciparum* HSP70 antibody at 1:300 and DAB-chromogen

Immunohistochemistry using anti-*P. falciparum* HSP70 antibody revealed positive immunoreactions in liver, lung, bone marrow, adrenal gland, kidney, pancreas, thyroid, and brain in erythrocytes in capillaries and blood vessels and intravascular leukocytes (Fig. 3 insets A, B and C and Fig. 4). The antibody stained diffusely in the cytoplasm of the parasites in infected erythrocytes of various organs, and haemozoin was evident as a black pigment (Fig. 4). The staining intensity related to the parasite maturity with immature trophozoites staining light brown while mature parasites stained dark brown (Fig. 4D). The monocytes and Kupffer cells in the liver showed immuno-positive reactions (7 µm in diameter) most likely staining phagocytozed parasites (Fig. 4C).

### Bacteriology and PCR tests for avian chlamydiosis and pigeon paramyxovirosis

All the bacteriological and PCR tests to detect *Chlamydia psittaci* and PPMV-1 were done at the NRGK. *Salmonella*, avian chlamydiosis and pigeon paramyxovirosis tested negative. Bacteriology of lung and liver yielded less than twenty colonies of unspecific bacteria in the liver and less than ten colonies of unspecific bacteria in the lung. The Ziehl–Neelsen staining of liver, adrenal gland, thyroid gland, and the cranial mass on the trachea detected no mycobacteria. Instead, there were suspicious inclusions in most of the erythrocytes seen in the smears from the adrenal gland, suggestive of haemoparasites. Further material (organs, blood clot) was subsequently handed over to the Institute of Parasitology (see parasitology tests above).

### Follow-up

The other pigeons of the owner were reported to be in good health. The owner was advised to isolate wild/feral birds and test them before mixing with his pets. Adoption of wild birds is not advised unless approved by veterinary authorities. Wild birds may harbour diseases that are transmitted to pets and/or to humans. It is also unadvisable to move birds between geographical borders, as this may spread diseases between regions. In January 2024, another dead pigeon was submitted to the NRGK by the same owner. Upon necropsy, the bird was emaciated. The gizzard contained two broken paperclips and the lining of the gizzard was pierced, causing ulcers. Upon screening for haemoparasites, the PCR was negative. A small study screening 22 city pigeons from Zürich, Switzerland, for *Plasmodium* spp., revealed only two birds positive in the lung and liver samples for *Haemoproteus* spp. STRURA03. These samples were not histologically analysed further.

## Discussion

This case describes avian malaria infection in a feral-pet pigeon, as confirmed by an extensive diagnostic work-up. The major finding in this study is *P. relictum* in a naturally infected wild bird confirmed by the presence of the parasite in the blood as seen on Hemacolor-stained smears, histopathology, immunofluorescence assay, and immunohistochemistry. *P. relictum* GRW11 lineage has been isolated before in different bird species including birds from the order Passeriformes (passerines), Sphenisciformes (penguins), Phoenicopteriformes (flamingos) and Strigiformes (owls) [25, 26, 36]. This is the first time this lineage has been reported in a pigeon (Columbiformes). Information on the pathogenicity of this lineage in natural infections is lacking. Experimental infections of GRW11 in canaries have reported mild infection with low parasitaemia during the acute phase, in contrast to *P. relictum* SGS1 which is known to cause high parasitaemia in canaries [37].

In the case presented here, the pigeon infected with *P. relictum* GRW11 had high parasitaemia and evidently  all erythrocytic stages of the parasite were present. The pigeon presented most likely died due to severe anaemia in the acute phase of infection, with high intraerythrocytic multiplication of the parasite, leading to erythrocyte lysis. This is corroborated by histopathology where in all organs sectioned, infected erythrocytes were detected and haemozoin pigment was seen in erythrocytes in blood vessels. When the rate of erythropoiesis cannot compensate for the erythrocyte loss through destruction, anaemia is inevitable [38]. Hill CM [39] reported severe anaemia due to reduced number of erythrocytes as a proximate cause of death in pigeons infected with *P. relictum*.

Pulmonary oedema and hydropericardium were evident on the gross evaluation of the bird. These changes are often seen in avian malaria infections [40]. Infection with *Plasmodium* triggers inflammatory infiltrates of leukocytes (i.e., monocytes, lymphocytes or heterophils), and these were noted in various organs [41, 42]. There was evidence of erythrophagocytosis in monocytes and macrophages with haemozoin pigments seen in the lung and liver indicative of an acute infection. This has been reported in previous studies with avian, human, and rodent malaria infections [26, 43]. The role of monocytes during infection is to phagocytose infected erythrocytes and control parasite burden, trigger antigen presentation and release cytokines. Studies have shown that phagocytosed haemozoin may impair monocyte and macrophage function and reduce the hosts’ ability to mount an immune response [43]. This factor may have contributed to the bird succumbing to infection.

Exoerythrocytic meronts (phanerozoites) were not seen in the examined organs. These parasitic forms are mostly known to originate from natural or sporozoite inoculated infections [28]. Phanerozoites may be differentiated from the phagocytosed erythrocytic meronts that we observed in this study by their lack of haemozoin pigment. In *P. relictum* infections, exoerythrocytic meronts are limited once the blood infection is established [28, 44]. Recent publications have reported the absence of phanerozoites in *P. relictum* infections in wild and experimental birds and some authors suggest that some strains of *P. relictum* may not develop phanerozoites altogether [20, 37, 45]*.* In canaries, phanerozoites were observed in endothelial cells of brain capillaries [38]. However, in a more recent study, these parasite stages were not detected in experimentally infected canaries [45]. Farmer JN and Moore AK [46] reported death in ten out of 38 *P. relictum* experimentally infected pigeons during the course of infection. In four of the ten dead birds, phanerozoites were observed mostly in the brain and some in the liver and spleen. In another study on *P. relictum*-infected puffins, phanerozoites have been observed in two out of three infected birds, in the liver, spleen, kidney, lung and heart [26]. These studies suggest that the development of exoerythrocytic parasite forms may be dependent on the parasite strain and/or the avian host infected. More studies are required to understand how the interaction between specific bird hosts and parasite strains influences the formation of these parasitic stages.

The physiological state of the host during infection plays a major role in determining infection outcome. For example, hosts in poor health may be predisposed to infectious diseases due to immunodeficiency [47]. The pigeon presented had a peri-bulbar tumour and surgery to remove it a few weeks before succumbing to avian malaria. Furthermore, on histopathology, there was evidence of a thyroidal neoplasm. These factors may have contributed to the demise of the bird. Our PCR results showed a coinfection with *Leucocytozoon* spp. AEMO02 in the adrenal gland. This lineage was first reported in feral pigeons in southern Italy and in a cinereous vulture(*Aegypius monachus*) in Spain [10]. However, the pathogenicity of this lineage is not described. Exoerythrocytic meronts of *Leucocytozoon* are reported to be found in hepatocytes, tubular cells of the kidney, macrophages of the reticuloendothelial cells as well as in endothelial cells of capillaries [48]. Analysis of impression smears and organ histology did not show any gametocytes or exoerythrocytic stages (meronts or megalomeronts) of *Leucocytozoon*. The fact that only one organ was PCR-positive may be suggestive of a previous or new infection with low parasitaemia that may only be detected by PCR. However, these scenarios could not be confirmed in this study.

Coinfections of *Plasmodium* and *Leucocytozoon* are common in naturally infected hosts [10, 49]. Some authors have shown that presence of haemosporidian parasite infection in birds predisposes to coinfection with another haemosporidian parasites, as is seen in our case. Coinfections may affect the host fitness and the pathogenicity of the parasite species or lineage [37, 49]. This scenario may be true in the current case as a virulent *P. relictum* GRW11 infection may have masked an effect of the *Leucocytozoon* infection.

Diagnosis of exoerythrocytic and erythrocytic stages of avian *Plasmodium* parasites in necropsy cases is normally done by histological analysis of Hemacolor rapid/ Giemsa and Haematoxylin and Eosin-stained tissue sections. This poses a challenge as it requires personnel training to visualize and identify the parasite stages. Other authors have used chromogenic in-situ hybridization (CISH) with *Plasmodium*-specific probes to stain parasites in the tissues [45, 50]. Anti-*P. falciparum* HSP70 has been used for the detection of *P. falciparum* and *Plasmodium berghei* mosquito, hepatocyte and erythrocytic stages [51]. Evidently mature parasites (gametocytes and schizonts) showed an increased staining in IFA and IHC compared to immature ones (trophozoites). This may be difficult to interpret on IHC as cut sections of tissues are used and this may modify the appearance of the parasite staining. However, this finding agrees with previous studies that demonstrated through a quantitative comparison an increase in HSP70 expression during the blood cycle in mature *P. berghei* parasite stages [51, 52]. Here we used immunochemical staining techniques (IHC and IFA) with the anti-*P. falciparum* HSP70 antibody, and we could successfully demonstrate that this antibody cross-reacts with avian *P. relictum* and may be used to aid the diagnosis of avian malaria.

## Conclusion

While *P. relictum* infections in pigeons are less frequently reported, sporadic occurrences may be fatal. The pigeon presented died suddenly and the carcass was severely anaemic with pale organs and on gross pathology revealed hydropericardium and pulmonary oedema. The bird most likely died from severe anaemia due to high blood parasitaemia from an acute *P. relictum* GRW11 infection with moderate pneumonia and hepatitis. Infected erythrocytes in tissues and blood vessels of various organs were seen microscopically, but no exoerythrocytic meronts were seen. The study presents for the first time use of an anti-*P. falciparum* HSP70 antibody for use in immunostaining methods to detect *P. relictum* parasites in the blood. Necropsy analysis further revealed a *Leucocytozoon* coinfection and thyroid carcinoma in the bird. These conditions may have exacerbated the *Plasmodium* infection, resulting in the bird succumbing to infection. Further analysis of pigeons complemented previous studies and suggest that avian *Plasmodium* infections are uncommon in pigeons in Switzerland.

## Supplementary Information

Supplementary material 1. Figure S1. Histopathology by haematoxylin and eosin staining of various organs. A. Kidney panel showing infected red blood cells and monocytes with haemozoin pigment in a blood vessel. Insert shows an IHC image of a blood vessel with erythrocytes stained with *P. falciparum *HSP70 antibody. B. Pancreas blood vessel showing infected red blood cells, monocytes with engulfed infected erythrocytes and haemozoin. Inset shows the same view in IHC stained with *P. falciparum *HSP70 antibody. C. Brain blood vessel showing infected erythrocytes with haemozoin pigments and the corresponding IHC stained with *P. falciparum *HSP70 antibody. Scale bar images: 50 μm, Insets: 30 μm. D. Shows the thyroid overview at 10x magnification. Several masses in the thyroid are evidently thyroid carcinoma. Scale bar. 250 μm. E. Inflammatory infiltrates in the thyroidal tissues. F. Infected red blood cells in blood vessels and haemozoin pigment in erythrocytes and monocytes.  Inset: zoom in view depicting infected red blood cells with asterisk. Scale bar images E and F 50 μm, F inset 10 μm.

## Data Availability

Partial cytochrome b DNA sequences from *P. relictum* GRW11 and *Leucocytozoon* spp. obtained in this study were deposited with Genbank under accession numbers PQ197205 and PQ197206, respectively.
