# Supplementary figures and images for: Avian malaria in a feral-pet pigeon: a case report
Source: Malar J. 2024 Oct 2;23:294. doi: 10.1186/s12936-024-05116-5 (PMC11446001; doi:10.1186/s12936-024-05116-5)

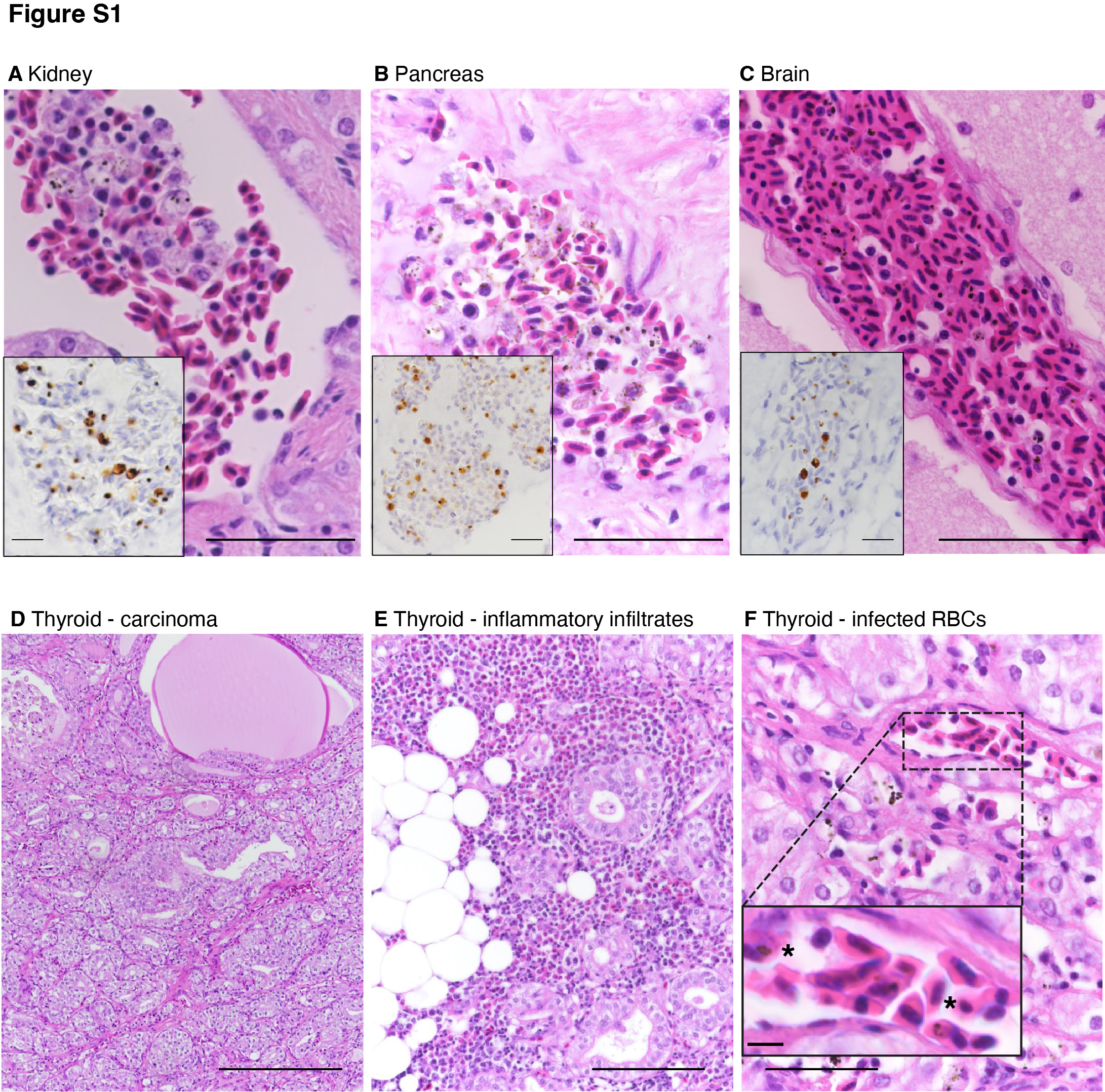

Supplement: Supplementary file 1 — Supplementary material 1. Figure S1. Histopathology by haematoxylin and eosin staining of various organs. A. Kidney panel showing infected red blood cells and monocytes with haemozoin pigment in a blood vessel. Insert shows an IHC image of a blood vessel with erythrocytes stained with P. falciparum HSP70 antibody. B. Pancreas blood vessel showing infected red blood cells, monocytes with engulfed infected erythrocytes and haemozoin. Inset shows the same view in IHC stained with P. falciparum HSP70 antibody. C. Brain blood vessel showing infected erythrocytes with haemozoin pigments and the corresponding IHC stained with P. falciparum HSP70 antibody. Scale bar images: 50 μm, Insets: 30 μm. D. Shows the thyroid overview at 10x magnification. Several masses in the thyroid are evidently thyroid carcinoma. Scale bar. 250 μm. E. Inflammatory infiltrates in the thyroidal tissues. F. Infected red blood cells in blood vessels and haemozoin pigment in erythrocytes and monocytes. Inset: zoom in view depicting infected red blood cells with asterisk. Scale bar images E and F 50 μm, F inset 10 μm. [file 12936_2024_5116_MOESM1_ESM.jpg]
